# Computed Tomography (CT) Perfusion in Abdominal Cancer: Technical Aspects

**DOI:** 10.3390/diagnostics3020261

**Published:** 2013-04-03

**Authors:** Martin Lundsgaard Hansen, Rikke Norling, Carsten Lauridsen, Eva Fallentin, Lene Bæksgaard, Klaus Fuglsang Kofoed, Lars Bo Svendsen, Michael Bachmann Nielsen

**Affiliations:** 1 Department of Diagnostic Radiology, Rigshospitalet, Blegdamsvej 9, Copenhagen 2100, Denmark; E-Mails: rikke.norling@regionh.dk (R.N.); cala@phmetropol.dk (C.L.); eva.fallentin@regionh.dk (E.F.); mbn@dadlnet.dk (M.B.N.); 2 Metropolitan University College, Radiography Education, Sigurdsgade 26, Copenhagen 2200, Denmark; 3 Department of Oncology, Rigshospitalet, Blegdamsvej 9, Copenhagen 2100, Denmark; E-Mail: Lene.baeksgaard.jensen@regionh.dk; 4 Department of Cardiology, Rigshospitalet, Blegdamsvej 9, Copenhagen 2100, Denmark; E-Mail: kkofoed@dadlnet.dk; 5 Department of Surgery C, Rigshospitalet, Blegdamsvej 9, Copenhagen 2100, Denmark; E-Mail: lars.bo.svendsen@regionh.dk

**Keywords:** CT Perfusion, cancer imaging, abdominal imaging, motion correction

## Abstract

Computed Tomography (CT) Perfusion is an evolving method to visualize perfusion in organs and tissue. With the introduction of multidetector CT scanners, it is now possible to cover up to 16 cm in one rotation, and thereby making it possible to scan entire organs such as the liver with a fixed table position. Advances in reconstruction algorithms make it possible to reduce the radiation dose for each examination to acceptable levels. Regarding abdominal imaging, CT perfusion is still considered a research tool, but several studies have proven it as a reliable non-invasive technique for assessment of vascularity. CT perfusion has also been used for tumor characterization, staging of disease, response evaluation of newer drugs targeted towards angiogenesis and as a method for early detection of recurrence after radiation and embolization. There are several software solutions available on the market today based on different perfusion algorithms. However, there is no consensus on which protocol and algorithm to use for specific organs. In this article, the authors give an introduction to CT perfusion in abdominal imaging introducing technical aspects for calculation of perfusion parameters, and considerations on patient preparation. This article also contains clinical cases to illustrate the use of CT perfusion in abdominal imaging.

## 1. Introduction

Recent advances in oncological treatment, with increasing focus on individualized treatment, calls for more advanced imaging modalities to stage diseases and evaluate treatment response. With the introduction of targeted drugs towards angiogenesis, there is furthermore a need of an image modality able to visualize changes in tissue perfusion before changes in tumor size. 

Computed Tomography (CT) Perfusion is a functional imaging modality to evaluate tissue vascularity [1,2]. It measures changes in tissue enhancement after contrast injection and tissue perfusion can thus be estimated with different kinetic models. Compared to other modalities, CT has the advantages of widespread availability and relatively low cost in addition to high spatial resolution [3].

CT perfusion is already well established in stroke evaluation [4], but is still considered a research tool regarding abdominal imaging. In the future, perfusion imaging could be added to already standardized CT protocols for various applications, such as diagnosis, staging, prognostic evaluation, and monitoring response to therapies. Further studies must be performed to establish consensus about scan protocols for standardized examinations. 

This article presents clinical cases and gives a brief overview of the application of CT perfusion in abdominal cancer. 

## 2. What is CT Perfusion?

Perfusion is the delivery of blood through the arterial system and capillaries to the tissue and it can be calculated by various techniques with different units. In large vessels, blood flow is measured as a velocity, but in the capillary bed perfusion is calculated as the volume of blood delivered to a volume of tissue at a given time (mL/min/100 mL) [5].

CT Perfusion requires administration of iodine contrast and dynamic image acquisition covering the structures of interest. In the absence of image artifacts, there is a linear correlation between the measured x-ray attenuation and the iodine concentration, which makes the mathematical modeling straightforward. By subtracting the initial unenhanced image, it is possible to create tissue attenuation curves voxel by voxel. It is important to take into consideration that iodine contrast is not a strict intravascular agent, so the enhancement represents both intravascular contrast and contrast in the extracellular space. 

## 3. Technical Aspects, Contrast Administration, Radiation Dose and Kinetic Models

Any modern CT scanner system can perform CT perfusion. The dynamic range is limited by the length of the detector rows, from 2 cm, and up to 16 cm at a 320-detector scanner with a fixed tube position. Some protocols have implemented a “jug-mode” or helical scans, where the detector row constantly moves back- and forth, thereby covering a larger area. The temporal resolution is reduced with the increased acquisition time for each volume and could introduce inaccuracies to the data set [6]. 

The radiation dose is a product of tube current, the number of images and the number of slices, *i.e.*, coverage along the z-axis. It has been previously reported that a CT perfusion protocol uses 1.5 times the radiation dose of a normal CT scan [7], but with wider coverage this number is probably higher and it varies depending on the selected protocol. The optimal tube voltage is 80–100 kV and the current-time product can be as low as 35 mAs. 

The optimal CT perfusion protocol is a trade-off between the amount of data points and the total radiation dose. With newer iterative image reconstruction methods, it is possible to reduce the radiation dose even more. Phantom studies have shown a significant reduction in image noise using iterative reconstruction [8,9], and Negi *et al.* [10] showed no differences in hepatic perfusion parameters when comparing filter back projection with iterative reconstruction.

The contrast should be administered as a bolus with a high flow (4–10 mL/s). The contrast volume should be low (40 to 50 mL) followed by a saline injection at the same rate. The high flow rate and relatively low bolus volume ensures favorable bolus geometry [11]. Use of contrast media with high concentration of iodine is preferred to increase the tissue enhancement [3]. In our experience with CT perfusion, the high flow rate does not give any extra discomfort to the patient. The placement of the intravenous access is also essential. In longitudinal studies the intravenous access should be consistently placed on the same side. In cases with close relations between the tissue of interest and the central veins, *i.e*., apical lung tumor, the intravenous access should be placed on the contralateral side to avoid streak-artifacts from the high contrast dose in the axillary vein. We have also observed backward flow to the jugular veins, which is important when analyzing CT perfusion studies of the neck. 

The post processing of the attenuation curves is based on different kinetic models and perfusion algorithms. A number of different algorithms are used. Commonly used models are Maximum Slope method (single compartment), Patlak method (double compartment) or deconvolution method. Depending on the target organ, some algorithms use one arterial input (pancreas, kidney, intestines), and others rely on dual blood supply (lungs, liver). The scanning protocol depends on both the organ of interest, the disease and the perfusion algorithm. For instance, maximum slope method requires a shorter acquisition time, more frequent data sampling and a higher contrast injection compared to the deconvolution method [12]. The single compartment model uses Fick’s principle and combines the intravascular and extravascular spaces into one. It provides estimates of arterial flow [13]. The double-compartmental model describes the exchange of contrast media between the intravascular- and extravascular space, also in the early phase (2 min), where there is no substantial drainage of contrast in the venous system. The model provides estimates of blood volume and the permeability of the capillaries [13]. Many of these models have been validated in animal experimental models [14,15,16,17] and to some extent also in humans, although overestimation of cerebral blood flow compared to ^15^O-H_2_O-PET in healthy subjects have been reported [18]. 

For more details on the kinetic models for perfusion, please refer to these references [5,13,19,20,21,22]. 

## 4. Patient Preparation

Earlier adoption of CT body perfusion required breath-hold during scan time to avoid imprecise measurements due to movement of tissue in and out of plane. With the advance of newer multi-slice scanners, the coverage in the z-axis is larger, thereby allowing the tissue of interest to be in the scanning area during shallow breathing. Kandel *et al*. [23] compared shallow breathing *versus* breath hold, and concluded that shallow breathing reduces the demand for motion correction. Also, for some patients it can be difficult to suspend their breathing during a CT perfusion examination, and then either taking a deep breath or slowly exhaling, both causing more movement distortion compared to shallow breathing [24]. Placing an abdominal strap around the patient’s abdomen will prevent the patient from deep breathing during acquisition. 

The planning of a CT perfusion examination can either be performed with an initial low-dose helical CT scan or guided by spine levels from previous studies. The perfusion study has to be done prior to any conventional contrast enhanced CT examination to avoid contrast contamination. 

For gastric tumors it is recommended to use water as oral contrast to get better tumor visualization. Likewise, artifacts from bowel peristalsis can be reduced by administering motility-inhibiting agents like hyoscine butylbromide or glucagon [25].

## 5. Motion Correction and Data Processing

**Figure 1:**
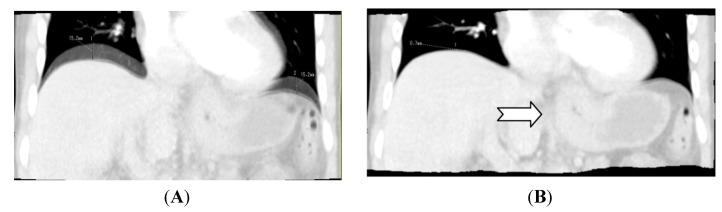
Illustrates the benefit of applying motion correction with abdominal CT Perfusion. (**A**) Image created by adding two scans into one image. There is a difference in the z-axis of 1.52 cm; (**B**) is created by adding the same scans as in (A), but after applying motion correction. The diaphragm in the two volumes is now well aligned. Note the tumor (arrow) in the gastro-esophageal junction is less blurry in (B) compared to (A) which makes perfusion calculations more accurate. (A) Without motion correction; (B) With motion correction.

The first step in data processing is motion correction. In-plane motion correction is possible with a rigid model, but with the advance of newer multi-slice scanners, motion reconstruction can also be done volumetric with correction in all planes. Correction can also be non-rigid taking organ deformation into account. Basically, registration models use differences in HU values to determine organ borders to align the registration. This can be troublesome for soft-tissue structures with small discrepancies to surrounding tissue. Non-rigid motion correction has been implemented in commercially available software semi-automatically, where the user picks a reference volume. Others have implemented a manual registration by predefining the areas of interest before registration [26]. This gives a more accurate registration, but is also more time-consuming. There have been no studies comparing semi-automatic registration and manual registration in regards to reproducibility of perfusion calculation (Figure 1).

Perfusion parameters are calculated on dedicated software, either directly on the scanner or a dedicated workstation. 

First, the arterial input is selected by drawing a region of interest (ROI) in the input artery. With upper abdominal imaging the ROI should be placed in the aorta. For lower abdominal imaging the iliac artery can be selected. It is important to make sure that the selected arterial ROI is inside the vessel on all series. Depending on the perfusion software, different parametric maps each representing a perfusion parameter are generated, which can be overlayed the CT scan. With volume scans and thin slices it is possible to do perfusion analysis in all planes, but the trade off is larger datasets and increase in image noise. Analysis can be done semi-quantitative by visually examining differences in perfusion or quantitative by placing ROIs in the targeted tissue. A concern regarding CT perfusion is reproducibility [27,28,29]. If the tumor is ill-defined or shows heterogenousity, there can be differences in defining the targeted tissue. This leads to previously observed inter- and intraobserver variation in perfusion calculation [30,31]. 

## 6. Clinical Examples with CT Perfusion

In oncology, there is a shift toward individualizing the patient’s treatment. In particular, drugs targeted towards tumor angiogenesis are increasingly used. CT perfusion visualizes the physiology in the vascular bed and some studies have shown a positive correlation between micro vessel density (MVD) and perfusion parameters [2,32,33], although others have failed to show this correlation [34,35]. CT perfusion has a potential role in both prognostic evaluation [36] and early assessment of treatment response [25]. Evaluating response after radiation therapy can sometimes be difficult, and it has been suggested that CT perfusion could help in dose planning [37]

**Figure 2:**
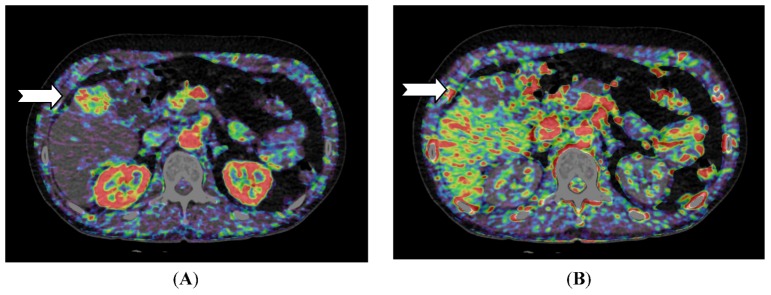
CT perfusion examination of a 54-year-old male with a solitary hepatocellular carcinoma (HCC) in the right liver lobe (arrow). (**A**) Perfusion shows high arterial blood supply and (**B**) lower portal blood supply compared with surrounding normal liver tissue (Images reconstructed with Vitrea 6.2, Vital Images A Toshiba Medical Systems Group).

**Figure 3:**
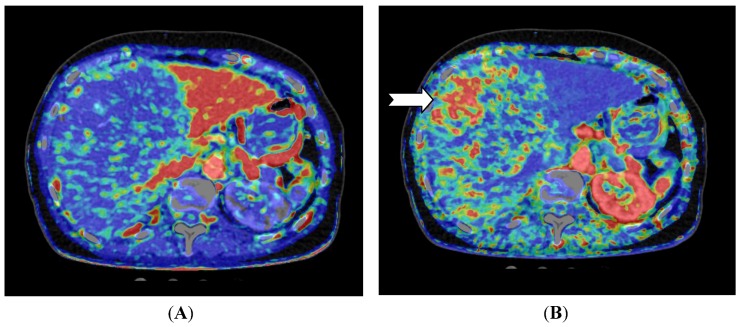
CT perfusion examination of a 77-year-old female after right-sided portal vein embolization prior to liver resection. The patient has a large HCC in the right liver lobe and segment 4. (**A**) Perfusion shows the portal flow, which is eliminated on the right side and elevated in the left liver lobe; (**B**) Perfusion index (Arterial Flow/Arterial Flow + Portal Flow). This index is low in the left side due to elevated portal flow, and the index is high in all of the embolized segments, but highest in the vascular part of the HCC (arrow) (Images reconstructed with Vitrea 6.2, Vital Images A Toshiba Medical Systems Group).

For liver lesions, Miles *et al*. [20] first described liver perfusion using CT in 1991 analyzing one 10 mm section. With multidetector CT the entire liver can be covered with a single rotation using a 320-detector row scanner. Applying a dual input—single compartment model provides estimates of both arterial and portal perfusion [38]. Since the first report, several different liver perfusion studies have published. CT perfusion of the liver has demonstrated higher arterial blood flow in cirrhotic patients [39,40] and CT perfusion has been used to characterize hepatocellular carcinoma and to examine for residual tumor after transarterial chemoembolization (TACE) (Figure 2 and Figure 3) [41].

**Figure 4:**
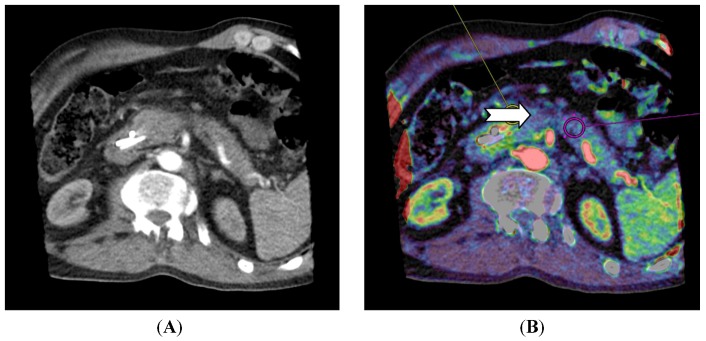
CT perfusion examination of a 63-year-old male with a pancreatic mass. The tumor has a neuroendocrine component and CT perfusion shows higher blood flow (110.2 mL/min/100 mL) (arrow) compared to the normal pancreatic tissue (83.7 mL/min/100 mL) (**B**). The patient has stents in the common bile duct (**A**) which causes image and perfusion artifacts in the pancreatic head. (Images reconstructed with Vitrea 6.2, Vital Images A Toshiba Medical Systems Group).

For pancreatic lesions, studies have shown that adenocarcinomas have a lower perfusion compared to normal pancreatic tissue [42,43]. For neuroendocrine tumors in the pancreas, D’ Assignies *et al.* [33] showed a higher perfusion and a positive correlation between blood flow and MVD (Figure 4). 

## 7. Conclusion

CT perfusion in abdominal cancer imaging is a novel method with promising results. With the advent of newer treatment options such as antiangiogenic drugs, there is a need for an image modality to visualize changes in perfusion prior to changes in size. There is no consensus on which CT protocol to use and published literature is based on small studies with different perfusion algorithms. 

The three cases described herein, illustrate the use of CT perfusion in abdominal pathology. 
